# Exome sequencing identified rare variants in genes *HSPG2* and *ATP2B4* in a family segregating developmental dysplasia of the hip

**DOI:** 10.1186/s12881-017-0393-8

**Published:** 2017-03-21

**Authors:** Sulman Basit, Alia M. Albalawi, Essa Alharby, Khalid I. Khoshhal

**Affiliations:** 1 Centre for Genetics and Inherited Diseases, Taibah University, Almadinah Almunawwarah, 30001 Saudi Arabia; 2 Department of Orthopedic Surgery, College of Medicine, Taibah University, Almadinah Almunawwarah, Saudi Arabia

**Keywords:** ATP2B4, Developmental dysplasia of the hip, Exome sequencing, Perlecan, HSPG2

## Abstract

**Background:**

Developmental dysplasia of the hip (DDH) is a common pathological condition of the musculoskeletal system in infants which results in a congenital and developmental malformation of the hip joint. DDH is a spectrum of pathologies affecting the infant hip ranging from asymptomatic subtle radiographic signs through mild instability to frank dislocations with acetabular dysplasia. A Saudi family with three affected individuals with DDH was identified and genetic analysis was performed to detect the possible genetic defect(s) underlying DDH in the affected members of the family.

**Methods:**

We performed whole genome genotyping using Illumina HumanOmni 2.5 M array and whole exome sequencing (WES) using Nextera Rapid capture kit and Illumina NextSeq500 instrument in four individuals of a family with DDH.

**Results:**

SNP data analysis did not identify any runs of homozygosity and copy number variations. Identity-by-descent (IBD) analysis on whole genome genotyping data identified a shared haplotypes on chromosome 1 in affected individuals. An analysis of the WES data identified rare heterozygous variants in *HSPG2* and *ATP2B4* genes in the affected individuals. Multiple prediction software predicted that the variants identified are damaging. Moreover, in silico analysis showed that HSPG2 regulates ATP2B4 expression using a variety of transcription factors.

**Conclusion:**

Our results indicate that there might be a functional epistatic interaction between HSPG2 and ATP2B4, and DDH in the family studied is due to a combined effect of both variants. These variants are also present in the asymptomatic mother suggesting that the variants in *HSPG2* and *ATP2B4* are incompletely penetrant. This study provides the first evidence of digenic inheritance of DDH in a family and extends the spectrum of genetic heterogeneity in this human disorder.

**Electronic supplementary material:**

The online version of this article (doi:10.1186/s12881-017-0393-8) contains supplementary material, which is available to authorized users.

## Background

Developmental dysplasia of the hip (DDH; MIM 142700) is a developmental disorder of the hip joint that results in an abnormal socket of the femoral head, ranging from instability, subluxation and complete dislocation of the hip joint, reduced joint function, and accelerated wear of the articular surface, which may result in early arthritis [1, 2]. Phenotypic variability has been observed in individuals affected with DDH.

The etiology of DDH is multifactorial, involving both genetic and environmental risk factors. Non-genetic risk factors include breech presentation, oligohydramnios, and primiparity [3]. Families with members affected with DDH has been reported [4–8]. This provides supports to the argument that DDH has a strong genetic basis [9–12]. Linkage analysis, exome sequencing and case-control association studies have so far identified several loci/genes associated with DDH [13]. Although, DDH is observed as an isolated condition in majority of patients with unilateral or bilateral hip involvement, it can be associated with other conditions such as clubfeet, renal malformations and cardiac anomalies [14].

An incidence of 1.5 - 20 cases of DDH per 1000 live births has been described [15]. Though, in some countries including Italy, Japan and other Mediterranean countries the prevalence of DDH is higher [16]. The variations in incidence rate is due to differences in diagnostic methods and the timing of evaluation of DDH affected individuals [17]. In the Middle East, few hospital based studies have shown an incidence rate of 3.17 - 3.50 per 1000 live births [18–20].

Genetic risk factors for DDH have mainly been searched for through case-control studies which have identified several candidate genes associated with bone and joint biology [4, 6, 8, 20–24]. These includes *GDF5, TBX4, ASPN, TGFB1* and *IL6* genes. However, no firm genotype-phenotype relations have been established for DDH yet.

Linkage analysis based on whole genome genotyping data has detected several chromosomal loci linked with DDH phenotype in extended pedigrees [6, 8, 20]. However, until now, studies presenting results from genetic analysis of such large families segregating DDH are very limited. So far, only two genes of interest were identified from such analyses. Feldman et al [2] identified a variant in *CX3CR1* shared by all DDH affected members of a 4-generational family. More recently, a mutation in the gene *UFSP2* was found to follow an autosomal dominant inheritance pattern with reduced penetrance (estimated at 80%) in a family from South-Africa with Buekes hip dysplasia [25].

The paucity of genetic information on DDH patients from Middle East emphasizes the need for comprehensive genomic studies to identify the candidate gene(s) in this population. We performed whole genome single nucleotide polymorphism (SNP) genotyping and whole exome sequencing (WES) in a Saudi family with three individuals showing DDH. We identified probably pathogenic heterozygous variants in *HSPG2* and *ATP2B4* genes in the affected individuals. Our data showed that the genetic variants underlying DDH are not completely penetrant.

## Methods

**Fig. 1:**
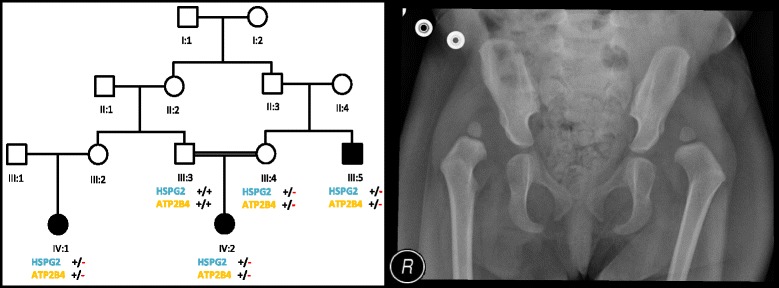
Pedigree drawings of the family with Developmental Dysplasia of the Hip (DDH). Open squares and circles represent unaffected males and females, respectively. Filled squares and circles represent affected individuals. Double lines between symbols are representative of consanguineous unions. **+/+** indicate wild type homozygote, while **+/-** indicate heterozygotes (**a**). Anterio-posterior radiographs of the pelvis of an affected individual (IV:2) showing bilateral hip dislocation with bilateral acetabular dysplasia (**b**)

Genetic analysis of 5 individuals (III:3, III:4, III:5, IV:1, IV:2) including 3 affected members (III:5, IV:1, IV:2) in a single family of Saudi origin (Fig. 1a) showing an isolated form of DDH was carried out. All affected individuals in the family were clinically and radiologically examined by a Pediatric Orthopedic Surgeon. Permission to undertake the study was obtained from the Research Ethics Committee (REC) of Taibah University. An informed consent for genetic evaluation was obtained from all participants. The blood samples from 5 individuals were obtained in EDTA tubes. Genomic DNA was isolated using QIAamp DNA mini kit. Concentration and quality of DNA was assessed by Nanodrop spectrophotometer (Green BioResearch, Baton Rouge, LA 70808, USA) and Qubit fluorometer (ThermoFisher Scientific Inc).

### Sanger Sequencing of Known Genes Associated with DDH

The family members were first tested for mutations in the known genes previously associated with DDH. The genes sequenced included asporin (*ASPN*) at chromosome 9q22.31, chemokine receptor 1 (*CX3CR1*) at chromosome 3p22.2, dickkopf WNT signaling pathway inhibitor 1 (*DKK1*) at chromosome 10q21.2, growth differentiation factor 5 (*GDF5*) at chromosome 20q11.22, homeobox B9 (*HOXB9*) at chromosome 17q21.32, homeobox D9 (*HOXD9*) at chromosome 2q31.1, pappalysin 2 (*PAPPA2*) at chromosome 1q25.2, and transforming growth factor, beta 1 (*TGFB1*) at chromosome 19q13.2.

### Whole genome SNP genotyping

Illumina HumanOmni 2.5 M SNP array was used for whole genome SNP genotyping. A total of 200 ng genomic DNA from four members (III:3, III:4, III:5, IV:2) of a family was used as a starting material. Briefly, 0.1 N NaOH was used for DNA denaturation and whole genome was amplified out with Random Primers Mix (RPM) using Multi Sample Master Mix (MSM). Enzymatic fragmentation of the amplified DNA was carried out using Fragmentation Mix (FMS) followed by precipitation using Precipitation Mix 1 (PM1) and 2-propanol. Fragmented DNA was hybridized to BeadChip by denaturing the sample and dispensing 35 ul of the sample onto the BeadChip section followed by incubation for 18 hours at 48 °C in the hybridization oven. Single base extension on BeadChips was performed after washing and staining. Single base extension reaction incorporates labeled nucleotides into the extended primers. Scanning of BeadChips was performed in Illumina iScan using iScan control software. Illumina GenomeStudio software and HomozygosityMapper were used to call loss of heterozygous (LOH) regions while the Illumina Genome Viewer incorporated in GenomeStudio was used to detect copy number variations (CNVs) in the genome. Identity-by-descent (IBD) analysis was carried out using PLINK [26] to identify shared genomic regions.

### Whole exome sequencing (WES)

Four individuals (III:3, III:4, III:5, IV:2) were initially available for exome sequencing. Nextera Rapid Capture Exome kit was used for library preparation and exome enrichment. This kit captures 214,405 exons and splice site and covers 98.3% RefSeq. Cluster generation and DNA sequencing was performed on Illumina NextSeq500 instrument.

Briefly, 50 ng of DNA was fragmented enzymatically and tagged with adaptor sequences (tagmentation) followed by purification and amplification of the purified tagmented DNA. Resulting libraries were purified with magnetic beads and target regions were captured with whole exome oligos followed by PCR amplification of the enriched library. Quantification of enriched library was performed with Qubit fluorometer and library size distribution was measured with Agilent Bioanalyzer. Quantified DNA library was loaded on flow cell for subsequent cluster generation and sequencing was carried out with an Illumina NextSeq500 instrument. NextSeq500 generates bcl files for each of the four lanes. These files were converted to fastq files using BCL2FASTQ tool. BWA aligner incorporated in BaseSpace was used to align fastq files to the reference genome using the BWA-MEM algorithm. Variants were called using genome analysis tool kit (GATK). Illumina VariantStudio was used for annotation and filtration of the genomic variants (Additional file 1: Figure S1). Loss of heterozygosity (LOH) analysis was performed on exome data of individuals III:5 and IV:2.

Sanger sequencing was performed for variants of interest to confirm the variants discovered by WES. Genomic sequences of the *HSPG2* and *ATP2B4* genes, including exons, introns, 5′ untranslated region and 3′ untranslated region, were retrieved from the Ensembl genome browser (http://asia.ensembl.org/index.html). Primer-3 software (http://frodo.wi.mit.edu/primer3/) was used to design primers for PCR amplification of the variants and their flanking regions. BIOEDIT sequence alignment editor version 6.0.7 (Ibis Biosciences Inc., Carlsbad, CA, USA) was used for sequence alignment.

Sanger reads for known genes sequenced in this study, whole genome SNP genotypes and exome variant files are available upon request.

## Results

### Clinical description of patients

The manifestation of DDH is bilateral hip dislocation in both affected individuals. Proband (IV:2) is a 2 year old female with bilateral DDH, discovered after watching her gait during walking. Her pelvic radiograph showed bilateral hip dislocation with bilateral acetabular dysplasia (Fig. 1b). She was treated by staged bilateral open hip reduction and pelvic osteotomy.

Her 30 years old maternal uncle (III:5) was presented with the same phenotypic manifestation. A third affected individual (IV:1) was identified while interviewing elders of the family. All affected individuals are of normal stature and have no other associated abnormality.

### Whole genome genotyping data analysis

Sequencing data analysis of the known genes (*ASPN*, *CX3CR1*, *DKK1*, *GDF5*, *HOXB9*, *HOXD9*, *PAPPA2*, *TGFB1*) excluded their involvement in causing DDH in the present family.

**Fig. 2:**
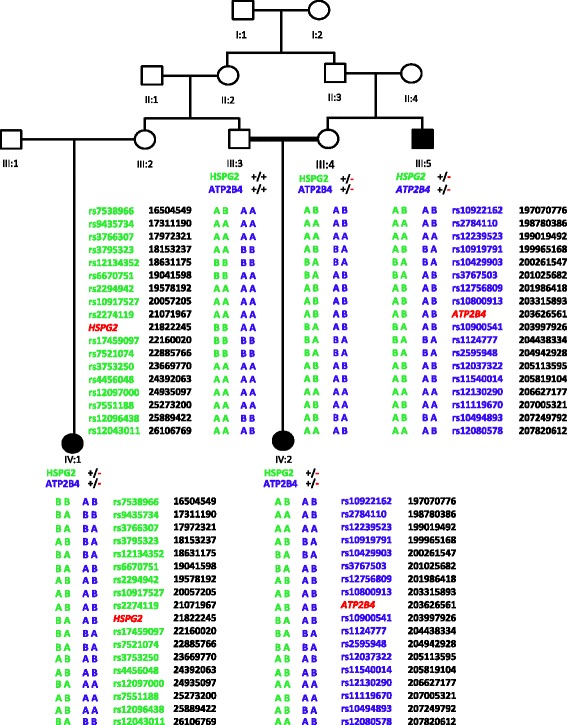
Haplotypes obtained from PLINK based IBD analysis using SNP genotyping data. All three affected individuals share haplotype carrying the mutant allele. Green color is used for *HSPG2* flanking haplotypes while blue color is used for *ATP2B4* flanking haplotypes

After excluding known genes, whole genome homozygosity mapping was carried out using Illumina 2.5 M BeadChip array. SNP genotypes were called by the BRLMM algorithm incorporated in Illumina GenomeStudio genotyping module. A call rate of more than 99% was obtained across the entire sample. Mapping order and physical and genetic distances of SNPs were obtained from Illumina. Analysis of SNP data to detect LOH was conducted using GenomeStudio software, HomozygosityMapper and AutoSNPa. However, no shared blocks of homozygosity were found in affected individuals. Moreover, Genome Viewer failed to find any shared pathogenic CNVs in the affected individuals. In order to identify genomic regions that are shared between affected individuals, genotyping data was subjected to IBD analysis using PLINK [26]. A shared haplotypes were detected on chromosome 1 in all 3 affected individuals (Fig. 2). The percentage of homozygosity ranged from 7-29% based on regions of homozygosity >10 Mb.

### Whole exome sequencing (WES) data analysis

WES was performed in the four individuals including the two affected (III:5, IV:2) and the two asymptomatic members (III:3, III:4) of the family. Average coverage achieved was between 65-92% (Table 1 and Table 2). The resulting variant call format (VCF) files contains on average 80,000 variants. These variants were filtered based on quality, frequency, genomic position, protein effect, pathogenicity and previous associations with the phenotype (Additional file 2: Figure S2).

**Table 1:** Pre-alignment statistics

Sample ID	Total Read Bases (bp)	Total Reads	Average read length	GC (%)	Q20 (%)	Q30 (%)	Mappable Reads (%)	Mean depth of target region (X)
III-3	11,149,473,250	84,775,682	131.52	44.7	78.0	64.3	95.6	65.0
III-4	15,035,517,174	107,770,286	139.51	44.0	77.6	63.6	95.8	79.2
III-5	15,498,839,720	113,685,720	136.33	44.1	81.2	68.0	96.7	92.8
IV-2	12,988,837,852	97,813,060	132.79	44.5	78.0	64.2	95.6	72.7

**Table 2:** Post-alignment statistics (SNP and INDELS)

Sample ID	Number of SNPs	Synonymous Variants	Missense Variants	Stop Gained	Stop Lost	Number of INDELs	Inframe INDELs	Present in dbSNP142 (%)	Het/Homo Ratio	Ts/Tv Ratio
III-3	48,829	9,692	8,542	67	33	3,208	252	98.3	1.1	2.4
III-4	51,488	9,632	8,457	64	26	3,545	237	98.1	1.0	2.4
III-5	56,735	10,333	9,066	79	25	4,959	267	97.7	1.2	2.4
IV-2	50,994	9,826	8,712	46	28	3,343	174	98.2	1.1	2.4

LOH analysis was performed on exome data of individuals III:5 and IV:2. However, no shared blocks of homozygosity were detected for these samples. In a first approach to find possible relevant variants in the affected members of this family, we looked for the previously reported variants in the *CX3CR1* and *UFSP2* genes; and then expanded the search for other potential candidate variants in the same genes. However, the described variants were not present in any of the family members nor any other candidate variants were found in these genes. Furthermore, WES reads for already known genes including *ASPN, CX3CR1, DKK1, GDF5, HOXB9, HOXD9, PAPPA2, TGFB1* were aligned and inspected manually for any deletion or duplication in these genes. No deletion or duplication in known genes were found.

In a second approach of finding candidate variants in the family, the exome data of the affected and the unaffected members were analyzed simultaneously. The pedigree was analyzed to establish the most likely inheritance pattern for a shared genetic cause of DDH in the family. De novo occurrence of candidate variants was not considered given the multiple affected family members with similar phenotypes and hence an expected shared genetic cause. X-linked inheritance was considered unlikely as there is an affected female in the family. An autosomal recessive inheritance could not be excluded based on the pedigree. However, homozygosity of a causal variant and compound heterozygosity in both affected family members, cannot be excluded.

Different filter settings were applied to look for potential candidate variants under an autosomal recessive inheritance model. More specifically, the analysis looked for (1) candidate variants present in homozygous state in IV:2, in heterozygous state in III:4, and present in homozygous or heterozygous state in III:5 [assuming at least a partially shared genetic cause of DDH in both affected members], (2) candidate variants present in homozygous state in III:5, in heterozygous state in III:4, and present in homozygous or heterozygous state in IV:2 [assuming at least a partially shared genetic cause of DDH in both affected members] and (3) candidate variants present in compound heterozygous state in IV:2, of which one variant is present in heterozygous state in both III:4 and III:5. In addition, another rare variant in the same gene would then be expected to be present in III:5. However, in accordance with the listed criteria here for an autosomal recessive model, no candidate variants were found.

Finally, an autosomal dominant inheritance with incomplete penetrance was considered. Interestingly, this inheritance pattern has been suggested in scientific literature for familial forms of DDH and is a valid option based on the pedigree. Under this model, individual III:4 (unaffected mother) would be expected to be an asymptomatic carrier of the candidate variant, and the affected individuals III:5 and IV:2 would be expected to both carry this candidate variant.

Similar to the family analysis performed by Feldman et al. [2], only rare variants were taken into account (allele frequencies below or equal to 1% in 1000G, ExAC and our in-house database of Saudi variants), and only variants located within genes (exonic and intronic) or promoter regions were considered. Furthermore, some quality parameters were taken into account including the depth of coverage (DP > 10) in at least 1 family member, the absence of allelic imbalance in at least 1 family member and good genotype quality (GQ > 20) in at least 1 family member.

Of the variants that came out of this filtering procedure, 59 were not present in dbSNP and were, as such, considered as 'new' variants. Genes in which these 59 variants are located are listed in Panel 1 and in supplementary table. Out of 59 variants, variants in 57 genes were present in all family members with no link to hip dysplasia. Only 2 genes (*HSPG2* and *ATP2B4*) were found to be linked to hip dysplasia or other skeletal or bone/joint abnormalities. The variants in these genes include heterozygous rare coding variants in the *HSPG2* (c.3328G > T) and *ATP2B4* (c.2264G > A) genes.

### Sanger validation

**Fig. 3:**
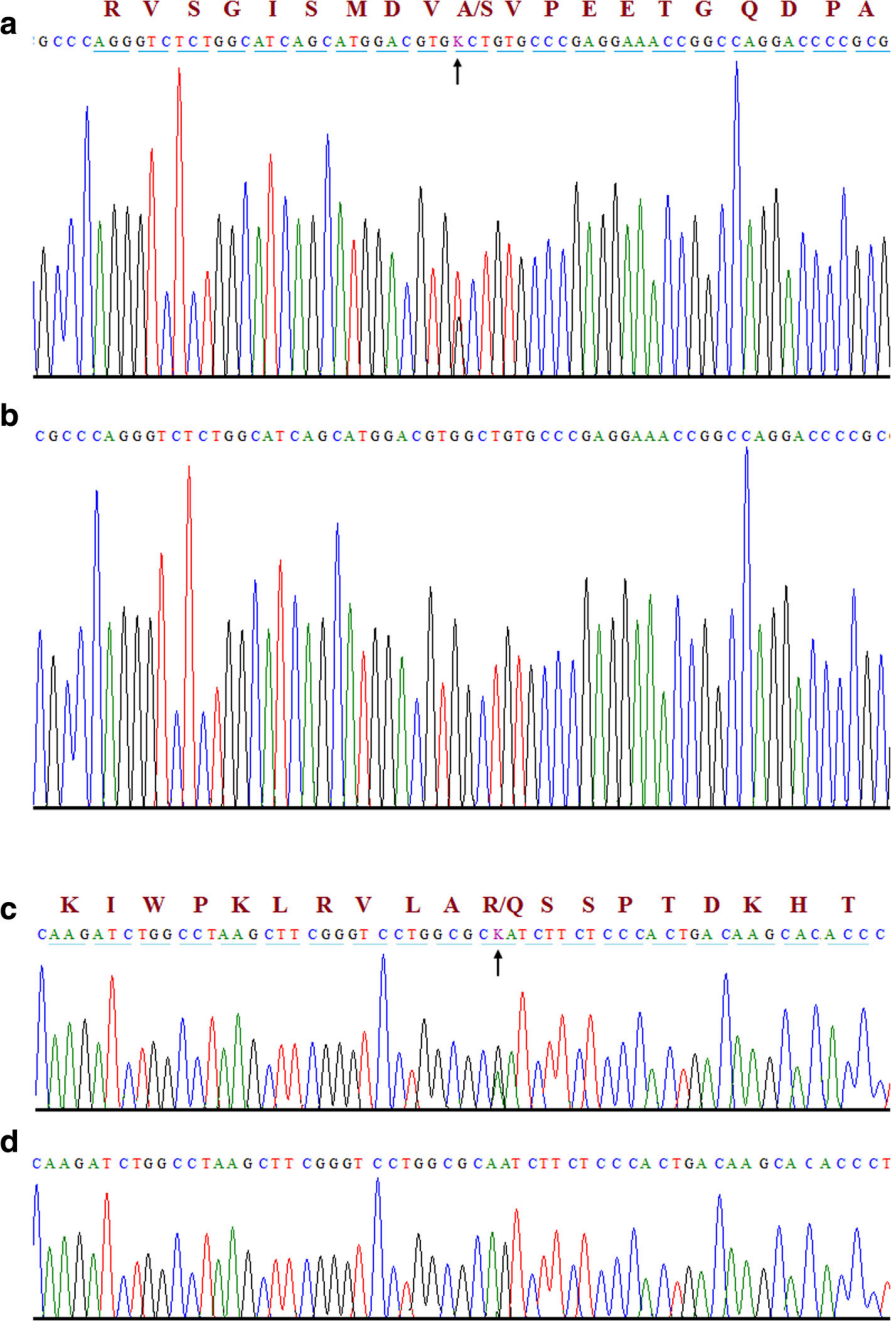
Sequence chromatograms of heterozygous variants identified in all three individuals of a family segregating DDH. The upper panel (**a**) represents the nucleotide sequences in all affected individuals in *HSPG2* (c.3328G > T) gene and the lower panel (**c**) represents the nucleotide sequences in all affected individuals in *ATP2B4* (c.2264G > A) gene. Panel **b** and **d** shows partial nucleotide sequence of the control individuals for *HSPG2* and *ATP2B4.* Arrow head indicates the variant site

The 59 variants were Sanger sequenced in all three affected individuals (III-5, IV-1, IV-2) and parents (III-3, III-4). All three affected individuals shared variants in *HSPG2* and *ATP2B4* genes only. The entire relevant coding exons of the *HSPG2* and *ATP2B4* genes were subsequently sequenced in all affected and unaffected subjects of the family. Variants identified through WES were also detected by Sanger sequencing (Fig. 3). A third affected individual (IV:1) was also screened using Sanger sequencing and was found positive for variants detected by exome sequencing.

### In silico analysis

**Fig. 4:**
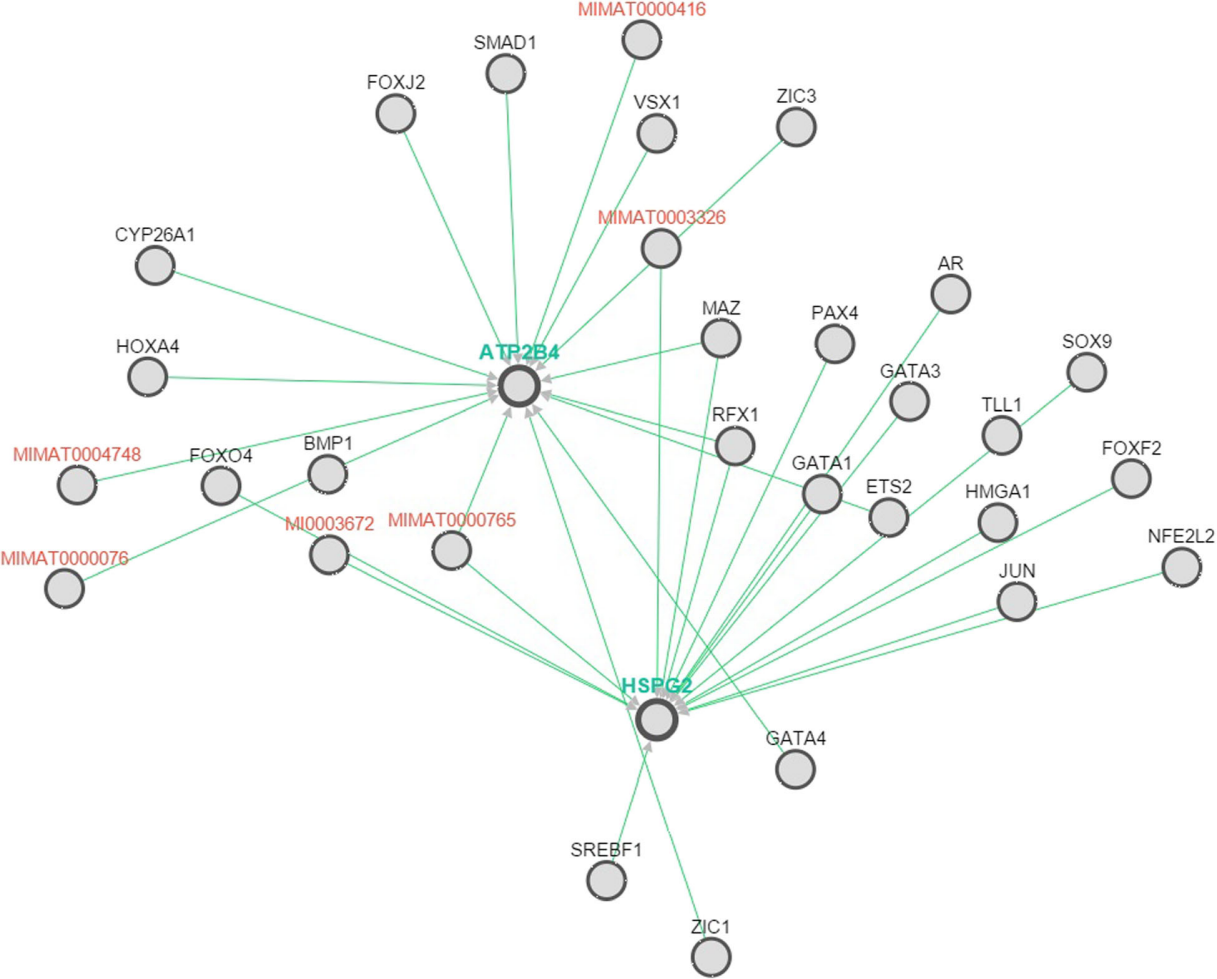
Pathway commons network visualizer (PCViz) was used to detect interaction between HSPG2 and ATP2B4 protein. No direct interaction between these two proteins were detected. However, HSPG2 indirectly controls expression of ATP2B4 via variety of transcription and regulatory factors including GATA1, MAZ and RFX1. Green lines between genes shows control of expression. Genes encoding miRNA are shown in red while transcription factors are shown in black text

Variants in HSPG2 and ATP2B4 occur at highly conserved residues (Table 3) and are predicted to be deleterious by the majority of bioinformatics results (Table 4). Moreover, I-Mutant software (used for prediction of protein stability upon single point mutation) predicted the mutant protein as less stable [27]. Furthermore, we used DUET (predicting effects of mutations on protein stability via an integrated computational approach), SDM (predicting effects of mutations on protein stability and malfunction) and mCSM (predicting the effect of mutation in protein using graph based signatures) for prediction of effect of mutation on the protein and found that these variants are indeed destabilizing [28–30]. Pathway commons network visualizer (PCViz) was used to detect interaction between HSPG2 and ATP2B4 protein [31]. No direct interaction between these two proteins was detected. However, HSPG2 indirectly controls expression of ATP2B4 via variety of transcription and regulatory factors including GATA1, MAZ and RFX1 (Fig. 4).

**Table 3:** Multiple sequence alignment for variants in HSPG2 (p. Ala1110Ser) and ATP2B4 (p. Arg755Gln)

Species	Ensembl ID for HSPG2	Alignment
Human	ENST00000341360	
Mutant		
Mmulatta	ENSMMUG00000014828	
Fcatus	ENSFCAG00000002965	
Mmusculus	ENSMUSG00000028763	
Ggallus	No Homologue	
Drerio	ENSDARG00000076564	
Xtropicalis	ENSXETG00000017911	
Species	Ensembl ID for ATP2B4	Alignment
Human	ENST00000341360	
Mutant		
Mmulatta	ENSMMUG00000021116	
Fcatus	ENSFCAG00000000870	
Mmusculus	ENSMUSG00000026463	
Ggallus	ENSGALG00000003601	
Drerio	ENSDARG00000044902	
Dmelanogaster	FBgn0259214

**Table 4:** Rare heterozygous exome variants [HSPG2 (c.3328G > T; p.Ala1110Ser) and ATP2B4 (c.2264G > A; p.Arg755Gln) identified in all affected individuals with developmental dysplasia of the hip

Gene	Variant Type	Global Minor Allele Frequency^a^	PolyPhen2	SIFT	Mutation Taster	Mutation Assessor	CADD	GERP++	PhastCons	SiPhy	Phylop	VEST3
HSPG2	Missense	0.0%	0.999	0.01	1.0	2.8	26.2	5.44	0.84	10.23	0.83	0.55
ATP2B4	Missense	0.0%	0.997	0.0	1.0	4.6	35.0	5.2	1.0	18.36	0.95	0.92

^a^1000 Genome, Exome Aggregation Consortium, 64 exome sequences from Saudi individuals

## Discussion

Exome variants were filtered considering an autosomal dominant inheritance with incomplete penetrance. Interestingly, this inheritance pattern has been suggested in scientific literature for familial forms of DDH and is a valid option based on the pedigree [2, 7, 25]. This model explains why the mother (III:4) is an asymptomatic carrier of the candidate variant, while the affected individuals IV:2 and III:5 are carrying these candidate variants.

A novel missense variant in *HSPG2* was detected in heterozygous state in both affected family members (IV:2 and III:5) and in the unaffected mother (III:4) of IV:2. The unaffected father does not carry this variant. Similarly, a novel missense variant in *ATP2B4* was detected in heterozygous state in the affected family members (IV:2 and III:5) and in the unaffected mother (III:4) of IV:2. The unaffected father does not carry this variant. Hence, the segregation of these variants is in accordance with an autosomal dominant inheritance with incomplete penetrance.

*HSPG2* encodes for the perlecan protein, a multi domain proteoglycan that cross-links extracellular matrix components and cell-surface molecules, which is essential for many biological activities. It has been shown that perlecan protein is strongly associated with musculoskeletal development, both in mouse and in human, making it a potential candidate gene for skeletal phenotypes such as DDH [32, 33]. Loss of function mutations in *HSPG2* results in a musculoskeletal phenotype in both mouse and humans. For instance, mutations in *HSPG2* gene can cause the autosomal recessive conditions Schwartz-Jampel syndrome type 1 (SJS1) and Silverman-Handmaker type of dyssegmental dysplasia. SJS1 is an autosomal recessive disease characterized by permanent myotonia and skeletal dysplasia, resulting in reduced stature, kyphoscoliosis, bowing of the diaphyses and irregular epiphyses. Other symptoms may include eye abnormalities such as cataract, which is also often associated with myotonic dystrophy [34]. Dyssegmental dysplasia Silverman-Handmaker type (DDHS) is an autosomal recessive skeletal dysplasia characterized by marked differences in size and shape of the vertebral bodies (anisospondyly) and short-limbed dwarfism. There are two recognized types: the severe, lethal DDSH (or lethal Kniest-like syndrome) and the milder Rolland-Desbuquois form. Individuals with DDSH also show flat face, micrognathia, cleft palate and reduced joint mobility. Moreover, they have an encephalocoele [35]. Besides these two Mendelian inherited conditions, a missense mutation in *HSPG2* (p.Asn786Ser) has recently been linked with idiopathic scoliosis based on exome sequencing analysis of a multigenerational family of European ancestry with familial scoliosis. Interestingly, segregation analysis in this family also suggested an autosomal dominant inheritance pattern with incomplete penetrance [36].

The variant detected here causes an alanine to serine (p.Ala1110Ser) missense substitution within laminin IV type A2 domain of the protein, which might affect protein interactions. *HSPG2* variation has been linked to hip dysplasia in both human and mice. Hip dysplasia can be present in patients with SJS1 [36]. In mice, a specific mutant *HSPG2* strain (C1532Yneo) was described to have reduced body weight and to develop chondrodysplasia and hip dysplasia [37].

*ATP2B4*, also known as PMCA4, encodes the plasma membrane calcium-transporting ATPase 4 enzyme, which is involved in catalyzing the hydrolysis of ATP, coupled with the transport of Ca^2+^ out of the cell. PCMA proteins including PCMA4 were suggested to play important roles in the regulation of bone homeostasis in both mice and humans by modulating calcium signaling in osteoclasts [38].

Mutations in *ATP2B4* have only recently been linked to a disease phenotype. More specifically, a specific missense mutation (c.803G > A, p.R268Q) was shown to cause autosomal dominant familial spastic paraplegia in a Chinese family [39]. Further studies on the detected mutation revealed functional changes in calcium homeostasis in human neuronal cells, suggesting that calcium dysregulation may be associated with the phenotype in that family [40].

The variant detected here causes an arginine to glutamine (p.Arg755Gln) missense substitution within a cytoplasmic domain of the ATP2B4 protein.

Based on *in silico* predictions, the altered residues of HSPG4 and ATP2B4 are conserved (Table 3), and the detected changes are predicted to have functional effects at the protein level (Table 4).

## Conclusions

In conclusion, these variants are reported based on their rarity in the general population (absent from 1000G, ExAC and dbSNP), in accordance with an autosomal dominant inheritance with incomplete penetrance shown by the family pedigree and the association of other *HSPG2* mutations with several skeletal phenotypes including hip dysplasia in human and mice points towards the role of *ATP2B4* in bone formation through modulation of calcium signaling.

A significant enrichment of the detected variants here or other rare *HSPG2* and *ATP2B4* variants in independent cohorts of DDH patients could provide a further evidence of the role of *HSPG4* and *ATP2B4* genes in general or the detected variants here in particular in the DDH phenotype.

Pathway analysis identified indirect interaction between HSPG2 and ATP2B4 proteins. HSPG2 regulate the ATP2B4 expression via a variety of transcription factors including GATA1, RFX1 and MAZ (Fig. 4). The genotype-phenotype relationship may be the combined effect of both mutations in each gene. Epistatic relationship between *HSPG2* and *ATP2B4* genes remain speculative until functional studies are performed.

This study provides the first evidence of a digenic inheritance in DDH in a family and extends the spectrum of genetic heterogeneity in this human DDH. *in vitro* functional studies are required to understand the mechanism underlying the role of epistatic interactions in DDH.

## Additional files

Additional file 1: Figure S1. Flow chart showing steps performed during generation of annotated variant file from raw data. (JPG 83 kb)

Additional file 2: Figure S2. Variant filtration process illustrating the exome filtering scheme in two affected individuals. Panel 1: Genes containing rare variants (1% in 1000G/ExAC/in-house database; absent from dbSNP) and in accordance with an autosomal dominant inheritance with incomplete penetrance (shared between affected samples III:5 and IV:2). ACOT8, ADCK1, AGMO, ANGPT4, ANKS3, ATP2B4, BARHL1, C12orf44, C18orf56, CACTIN, CCM2L, CEACAM4, CRISPLD2, CTAGE7P, CTSA, CTSE, DAGLB, DAZAP2, DCTN4, DENND1B, EDEM1, EMG1, FAM154A, FAM19A2, GSK3A, HIPK1, HMCN2, HNRNPUL1, HSPG2, INCENP, INTS1, IRG1, KCNIP4, KHSRP, LEPREL2, LPPR3, MAN1C1, MICALCL, MREG, MUC5B, NAV1, NEURL2, NPR2, NUCKS1, PCSK5, PDS5A, PLCG2, POMT1, PPL, PRDM7, PRKAB1, SEZ6L, SLC6A12, SLC7A5, SLIT2, ZCCHC8, ZNF335, ZNF648, ZNF780A. (JPG 128 kb)

## Abbreviations

ATP2B4: ATPase plasma membrane Ca2+ transporting 4
CGID: Center for genetics and inherited diseases
DDH: developmental dysplasia of the hip
HSPG2: heparan sulfate proteoglycan 2
MIM: mendelian inheritance in man
SNP: single nucleotide polymorphism
WES: whole exome sequencing
