# Supplementary figures and images for: Exome sequencing identified rare variants in genes HSPG2 and ATP2B4 in a family segregating developmental dysplasia of the hip
Source: BMC Med Genet. 2017 Mar 21;18:34. doi: 10.1186/s12881-017-0393-8 (PMC5361705; doi:10.1186/s12881-017-0393-8)

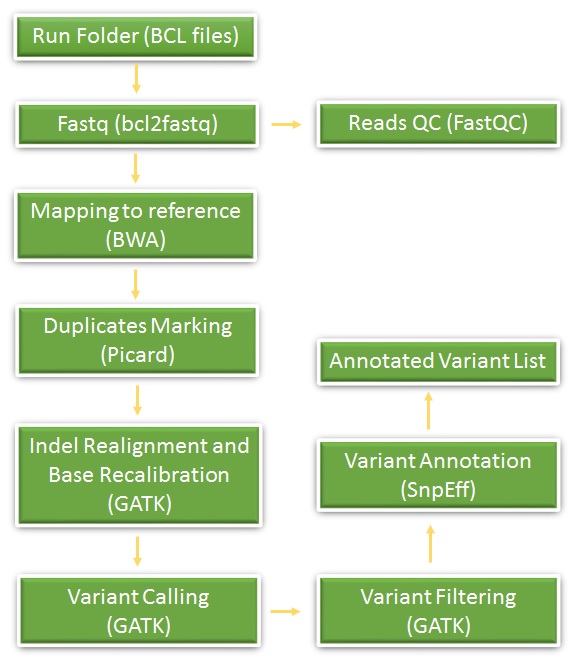

Supplement: Supplementary file 1 — Flow chart showing steps performed during generation of annotated variant file from raw data. (JPG 83 kb) [file 12881_2017_393_MOESM1_ESM.jpg]

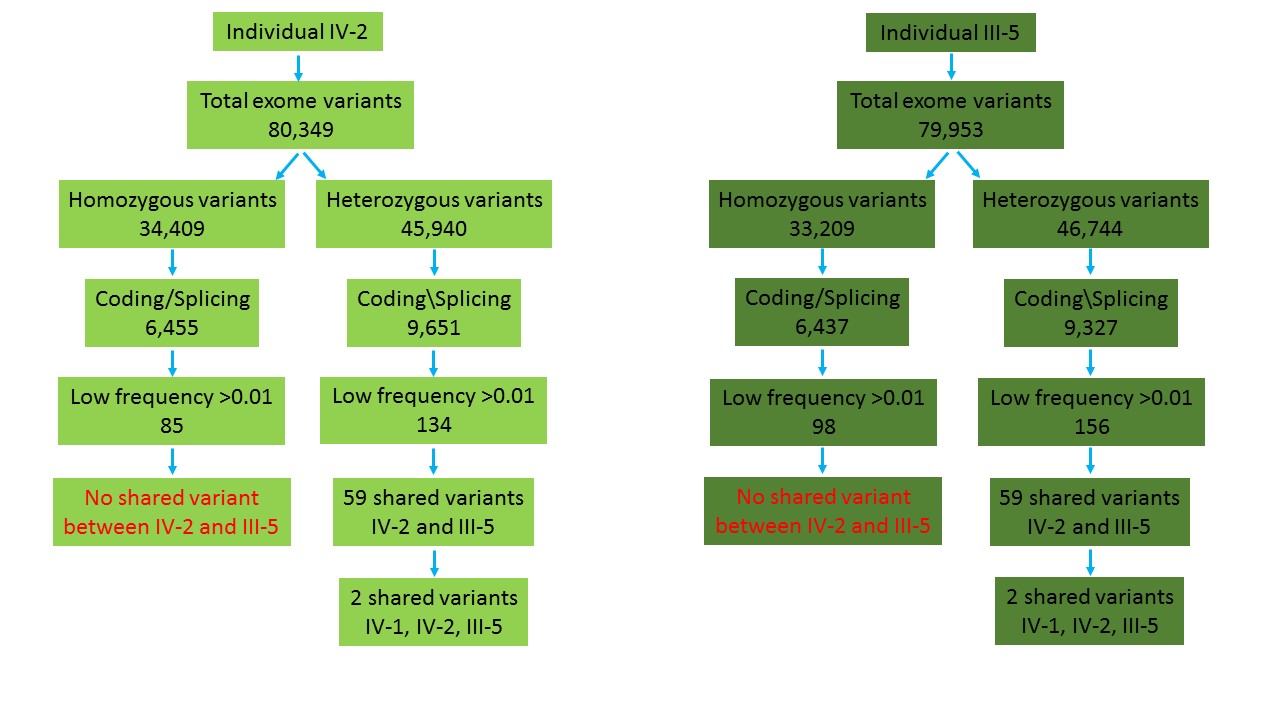

Supplement: Supplementary file 2 — Variant filtration process illustrating the exome filtering scheme in two affected individuals. Panel 1: Genes containing rare variants (1% in 1000G/ExAC/in-house database; absent from dbSNP) and in accordance with an autosomal dominant inheritance with incomplete penetrance (shared between affected samples III:5 and IV:2). ACOT8, ADCK1, AGMO, ANGPT4, ANKS3, ATP2B4, BARHL1, C12orf44, C18orf56, CACTIN, CCM2L, CEACAM4, CRISPLD2, CTAGE7P, CTSA, CTSE, DAGLB, DAZAP2, DCTN4, DENND1B, EDEM1, EMG1, FAM154A, FAM19A2, GSK3A, HIPK1, HMCN2, HNRNPUL1, HSPG2, INCENP, INTS1, IRG1, KCNIP4, KHSRP, LEPREL2, LPPR3, MAN1C1, MICALCL, MREG, MUC5B, NAV1, NEURL2, NPR2, NUCKS1, PCSK5, PDS5A, PLCG2, POMT1, PPL, PRDM7, PRKAB1, SEZ6L, SLC6A12, SLC7A5, SLIT2, ZCCHC8, ZNF335, ZNF648, ZNF780A. (JPG 128 kb) [file 12881_2017_393_MOESM2_ESM.jpg]
